# Study protocol on antimicrobial resistance burden, transmission dynamics, and therapeutic bacteriophages in livestock and exposed farming populations in Nagpur, India: An integrated One Health approach

**DOI:** 10.1371/journal.pone.0350919

**Published:** 2026-06-16

**Authors:** Amit R. Nayak, Jayshree Shukla, Sushrut Kulkarni, Rima Biswas, Nitin Kurkure, Megha Kaore, Sandeep Chaudhari, Urmi Bajpai, Krishnamurthi Kannan, SaravanaDevi Sivanesan, Sadanand D. Sontakke, Aliabbas Husain, Pankaj M. Kulurkar, Amit Bafna, Rajpal Singh Kashyap

**Affiliations:** 1 Dr. G.M. Taori Central India Institute of Medical Sciences (CIIMS), Nagpur, India; 2 Nagpur Veterinary College, Maharashtra Animal and Fishery Sciences University (MAFSU), Nagpur, India; 3 Acharya Narendra Dev College, University of Delhi, New Delhi, India; 4 Department of Biochemistry, University of Delhi, South Campus (UDSC), Benito Juarez Marg, Moti Bagh, New Delhi, India; 5 CSIR-National Environmental Engineering Research Institute (NEERI), Nagpur, India; PLOS: Public Library of Science, UNITED STATES OF AMERICA

## Abstract

The rise in antimicrobial resistance (AMR) is a severe public health threat worldwide. India bears a disproportionately heavy burden of this problem due to ample antimicrobial usage in both humans and animals and scarce integrated surveillance. Since humans, animals, and environmental reservoirs which can harbour resistant microorganisms interact very closely on farms livestock, these are considered critical hotspots for the emergence and dissemination of antimicrobial-resistant bacteria and resistance genes. This work presents a 36-month prospective longitudinal observational study protocol aimed at quantifying the burden and characterizing the transmission dynamics of a selected set of key bacteria, that are clinically significant and hence, pathogenic—*Escherichia coli*, *Staphylococcus aureus*, *Klebsiella pneumoniae*, *Streptococcus pneumoniae*, *Acinetobacter baumannii*, and *Pseudomonas aeruginosa*—alongside their AMRprofiles in livestock, farm-exposed human populations, and environmental reservoirs in Nagpur, India, within the framework of One Health. Seasonal sampling of milk, animal faeces, human stool, soil, wastewater, drinking water, and animal feed will be carried out on dairy farms located in urban, peri-urban, and rural areas. Pathogens will be isolated using standard microbiological techniques and characterized based on antimicrobial susceptibility by employing VITEK®2 and disc diffusion methods. At the same time, bacteriophages against multidrug-resistant isolates will be isolated, purified, and characterized through plaque assays, host-range analysis, electron microscopy, and whole-genome sequencing for their therapeutic potential evaluation. Additionally, metagenomic next-generation sequencing will be utilized on a select number of samples to comprehensively characterize the resistomes and diversity of phages. The research will provide detailed longitudinal data on the frequency and spread of AMR among human, animal, and environmental compartments, create a biobank of AMR isolates and lytic bacteriophages, and offer genomic clues to delineate phage-based treatments and well-informed mitigation strategies of AMR within the framework of One Health in India. The results will be made public through peer-reviewed articles, presentations at scientific meetings, and deposition of sequence data in open-access databases.

## Introduction

Antimicrobial resistance (AMR) is a critical global health challenge, undermining the effective treatment of infectious diseases. In India, infectious diseases contribute to a high mortality rate of 417 per 100,000 persons annually, with pneumonia alone accounting for approximately 410,000 deaths among children under five years, representing nearly a quarter of all child deaths in the country [1]. The rapid emergence and spread of resistant bacteria, including resistance to last-resort antibiotics such as carbapenems, has intensified the public health burden [2].

In the animal farming occupation populations, humans and livestock live in close proximity and share environmental resources, represent hotspots for the emergence and transmission of AMR bacteria and antimicrobial resistance genes (ARGs). Despite this, systematic investigations into AMR prevalence in livestock and associated human populations in India are limited, with most evidence derived from small, localized studies [3]. The unregulated use of antibiotics for therapeutic and non-therapeutic purposes in livestock, coupled with the lack of national surveillance systems for antibiotic usage or resistance, suggests that the contribution of the animal sector to the AMR burden is substantial yet largely unmeasured [4]. One of the most common clinical issues in dairy farming is mastitis, which may be subclinical or overtly symptomatic. Milk from mastitic animals has been shown to harbor diverse bacterial populations, including multidrug-resistant strains, highlighting livestock as a key reservoir of AMR bacteria [5].

Similarly, bacteriophages (phages) have re-emerged as a promising alternative to antibiotics for controlling multidrug-resistant pathogens. Phages are host-specific, self-replicating, environmentally sustainable, and generally safe for humans and animals. Advances in high-throughput screening and next-generation sequencing now allow systematic discovery, characterization, and genomic profiling of phages, providing a rational basis for their therapeutic application [6,7].

Despite significant technical advances in studying AMR and its transmission dynamics, no prior study in central India has integrated human, animal, and environmental AMR surveillance with therapeutic phage discovery under a One Health framework. The primary aim of this study is to assess the burden of key clinically significant pathogens—*Escherichia coli, Staphylococcus aureus, Klebsiella pneumoniae, Streptococcus pneumoniae, Acinetobacter baumannii,* and *Pseudomonas aeruginosa*—and to characterize their AMR profiles in animals, humans, and environmental samples from cattle farms in Nagpur, India. In parallel, the study seeks to isolate and characterize bacteriophages as potential alternative therapeutic agents. The secondary objectives are to investigate the diversity of the resistome across different reservoirs, evaluate AMR carriage in humans living in close contact with livestock, generate evidence for inter-species transmission of resistant bacteria and resistance genes, and examine the seasonal dynamics of AMR dissemination influenced by environmental factors.

## Methods

### Study design

**Fig 1:**
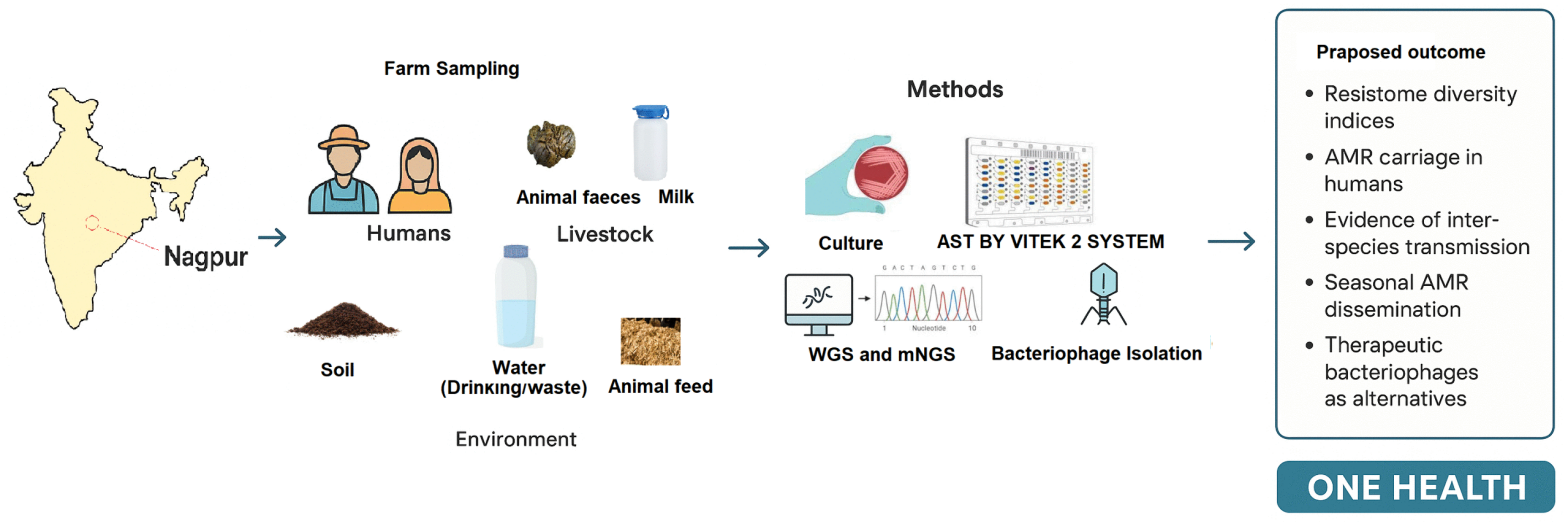
Overview of the One Health study in Nagpur depicting sampling from humans, livestock, and environment, followed by culture, AST, sequencing, and bacteriophage isolation methods.

This study will be a 36-month prospective longitudinal observational investigation aimed at understanding the prevalence and transmission dynamics of AMR and bacteriophage diversity across livestock, humans, and environmental reservoirs. It will be conducted across urban, peri-urban, and rural regions of Nagpur, India, to capture spatial and seasonal variations in AMR dissemination and microbial ecology. The longitudinal design will involve repeated sampling of animal, human, and environmental samples across three distinct seasons of the year, for evaluation of temporal changes in microbial populations, resistome, and phage communities, to understand the transmission dynamics. Overview of the One Health study, plan is given in Fig 1.

Permission to conduct sampling at livestock farms has been obtained from farm owners and management prior to sample collection. Environmental sampling, including soil, wastewater, drinking water, and animal feed from farm premises, was conducted with the consent of farm authorities and in accordance with institutional biosafety guidelines by CSIR-National Environmental Engineering Research Institute (NEERI). Similarly, animal sampling procedures were performed by the of Nagpur Veterinary College, Maharashtra Animal and Fishery Sciences University (MAFSU), with prior approval from the respective farm owners. No additional governmental permits were required for the collection of environmental samples within privately owned farm premises.

### Study status and timeline

At the time of manuscript submission, participant recruitment and sample collection have begun but remain ongoing. Recruitment of volunteers began on 30/03/2025 following institutional approvals and will continue as part of the planned longitudinal design. Participant recruitment and sample collection are expected to continue until the end of 2027. Data collection will therefore be completed by December 2027, after which final laboratory analyses and integrated data analysis will be conducted. No final analyses have been performed and no results have been generated or published at the time of submission. Study results are expected after completion of data collection and analysis in 2028.

### Study setting

Dairy farms (N = 5) with a minimum of 100 cattle will be selected for inclusion in this study. These farms represent diverse animal husbandry methods, agricultural practices, and environmental exposures in the Nagpur region. In addition to livestock sampling, associated human participants—including farmers, veterinarians, farm attendants, and family members in close contact with animals—will be included to study potential zoonotic transmission of key pathogens between animal and human. Environmental samples will include soil, wastewater, drinking water and feed from farm surroundings to assess environmental reservoirs of AMR and bacteriophage populations.

### Sample size

The sample size was estimated using the Rosoff tool (http://www.raosoft.com/samplesize.html) assuming an expected AMR prevalence of 50%, at 95.5% confidence level, and a 4.5% margin of error. Based on this calculation, approximately 480 human samples will be collected over the study period. From each farm, 12 human stool samples will be obtained every four months across eight sampling rounds. In parallel, livestock sampling will include six milk and six fecal samples per farm at each time point, while environmental sampling will consist of four samples (soil, water, feed, or wastewater) per farm at each time point, overall study sample size is depicted in Table 1. This systematic sampling strategy, conducted across three distinct seasons, is designed to capture temporal variability and ensure adequate statistical power to detect meaningful differences in AMR prevalence and bacteriophage diversity across human, animal, and environmental reservoirs.

**Table 1:** Sampling framework showing human, livestock, and environmental samples collected across farms and rounds for culture, Bacterial antibiotic sensitivity test, Whole Genome Sequencing, and metagenomics under proposed research study.

Sample Type	Sample type (Source)	Numberof sample/ Farm/Round	No. of Farms	Total Rounds (one round/4 month)	Estimated Samples Size	Analysis Planned
**Humans**	Stool (farmers, family, attendants, veterinarians)	12	5	8	480	Culture, AST, WGS, Metagenomics
**Livestock**	Milk (cattle)	6	5	8	240	
	Faces (cattle)	6	5	8	240	
**Environment**	Soil	1	5	8	40	
	Wastewater	1	5	8	40	
	Drinking water	1	5	8	40	
	Animal feed	1	5	8	40	

### Study population and sampling

#### Livestock sampling.

From each dairy farm, milk and fecal samples will be collected from six randomly selected animals every four months over a two-year period to enable longitudinal monitoring of microbial populations and AMR patterns.

Milk samples will be collected aseptically after thorough cleaning of teats with iodine or hypochlorite disinfectant, followed by wiping with clean cloth. The initial three to four streams of milk will be discarded to reduce contamination. Subsequently, 2–3 mL of milk will be collected from each teat into sterile, labeled vials, which will be immediately sealed and stored on ice. Additionally, pooled samples of approximately 10 mL will be collected from all teats into sterile containers. Samples will be transported in an icebox to the laboratory within 8–12 hours or stored at 4 °C and processed within 24 hours if immediate analysis is not possible. Fecal samples will be collected from the same animals using sterile rectal swabs containing Cary-Blair medium without any antibiotics. Samples will be properly labelled and transported under cold chain conditions to the laboratory. For all samples, metadata including date of collection, source, type of sample, animal identification, health and disease status, vaccination history, antimicrobial usage, and performance parameters will be systematically recorded.

#### Human sampling.

Human participants will include farm attendants, and the family members residing or working on the selected farms. Written consent will be acquired from all participants. Stool samples will be collected from consenting adults, only. Detailed metadata will be recorded for each participant, including demographic information (age, sex), comorbidities, prior antibiotic exposure, occupational history, dietary habits, and contact patterns with livestock and farm environments etc. For human stool collection, sterile wide-mouth containers will be provided to participants with instructions, and early morning stool samples (>10 g) will be collected, placed in sterile containers maintained at 4°C, and transported promptly to the laboratory.

#### Environmental sampling.

Environmental samples, including soil, wastewater, drinking water, and animal feed, will be collected quarterly from all participating farms to monitor microbial populations and potential contamination/transmission sources. Samples will be collected aseptically using sterile tools and containers, with approximately 50 grams of soil and 500 mL/1000 ml of wastewater/ drinking water collected per site. Feed samples will be taken representative of the animals’ diet. All samples will be immediately placed on ice and transported to the laboratory within 8–12 hours to preserve microbial viability. Upon arrival, samples will be homogenized and filtered as needed, prior to microbiological and molecular analyses.

### Enrichment of samples

Fecal/stool samples (3 g) will be homogenized in normal saline, centrifuged, and the pellet will be inoculated in 5 mL buffered peptone water (Oxoid). For enrichment, milk samples (1 mL) will be inoculated into 9 mL tryptone soya broth or MacConkey broth and incubated at 37 °C for 18–24 h. Soil or feed samples (10 g) will be suspended in saline or phosphate buffer, vortexed, centrifuged, and 1 mL supernatant will be inoculated into 9 mL MacConkey broth for incubation at 37 °C for 18–24 h. Dairy sewage water will be centrifuged, and 1 mL of the supernatant will be inoculated into 9 mL MacConkey broth followed by incubation at 37 °C for 18–24 h. Drinking water samples will be processed using a ten-fold dilution method, with the 10 ⁻ ⁴ dilution (1 mL) inoculated into 9 mL MacConkey broth and incubated at 37 °C for 18–24 h.

### Antimicrobial resistance screening

Targeted key pathogens, will be isolated from human, livestock, and environmental samples, at CIIMS, MAFSU, CSIR-NEERI Nagpur laboratory respectively using plating techniques on selective media and differential media such as HiCrome agar (Himedia India) (Table 2). Isolates will be confirmed using biochemical tests. Antimicrobial susceptibility testing and minimum inhibitory concentration (MIC) will be performed using VITEK®2 (Table 3). Isolates will be categorized as susceptible, intermediate, or resistant, providing a comprehensive AMR profile across study populations.

**Table 2:** Target bacterial pathogens were isolated on selective/differential culture media (as listed), and presumptive colonies were identified based on typical morphology.

Sr. No.	Pathogen	Selective/ Differential Media	Typical Colony Morphology
1	*Staphylococcus aureus*	HiCrome™ Staph Aureus Agar/ Baird-Parker Agar	Brown-black colonies (Baird-Parker); characteristic-colored colonies (HiCrome)
2	*Escherichia coli*	MacConkey Agar → EMB Agar	Pink lactose-fermenting colonies (MacConkey); metallic sheen colonies (EMB)
3	*E. coli O157:H7*	Cefixime–Tellurite Sorbitol MacConkey (CT-SMAC) Agar	Non-sorbitol-fermenting (grey/white) colonies
4	*Klebsiella pneumoniae*	HiCrome™ Klebsiella Selective Agar Base	Magenta-colored colonies
5	*Pseudomonas aeruginosa*	HiFluoro Pseudomonas Agar	Light amber opalescent/transparent gel-like colonies
6	*Streptococcus pneumoniae*	Blood Agar (with bile solubility test)	Mucoid colonies with alpha-hemolysis; soluble in bile
7	*Acinetobacter baumannii*	CHROMagar Acinetobacter	Red colonies

**Table 3:** Antibiotics tested for antimicrobial susceptibility profiling of target pathogens using VITEK®2.

Sr. No.	Pathogen	Gram Stain	Antibiotics Tested
1	E. coli	Negative	Amoxicillin–Clavulanic acid, Piperacillin–Tazobactam, Cefuroxime, Cefuroxime Axetil, Ceftriaxone, Cefoperazone–Sulbactam, Cefepime, Ertapenem, Imipenem, Meropenem, Amikacin, Gentamicin, Ciprofloxacin, Tigecycline, Fosfomycin, Colistin, Trimethoprim–Sulfamethoxazole
2	K. pneumoniae		
3	A. baumannii		
4	P. aeruginosa		
5	S. aureus	Positive	Beta-lactamase, Cefoxitin, Benzylpenicillin, Oxacillin, Gentamicin (high-level, synergy), Levofloxacin, Inducible Clindamycin Resistance, Erythromycin, Clindamycin, Linezolid, Daptomycin, Teicoplanin, Vancomycin, Tetracycline, Nitrofurantoin, Rifampicin, Trimethoprim–Sulfamethoxazole, Cefoxitin screen
6	S. pneumoniae		

## Phage isolation and characterization

### Isolation of bacteriophages

#### Bacteriophage lysate preparation.

**Fig 2:**
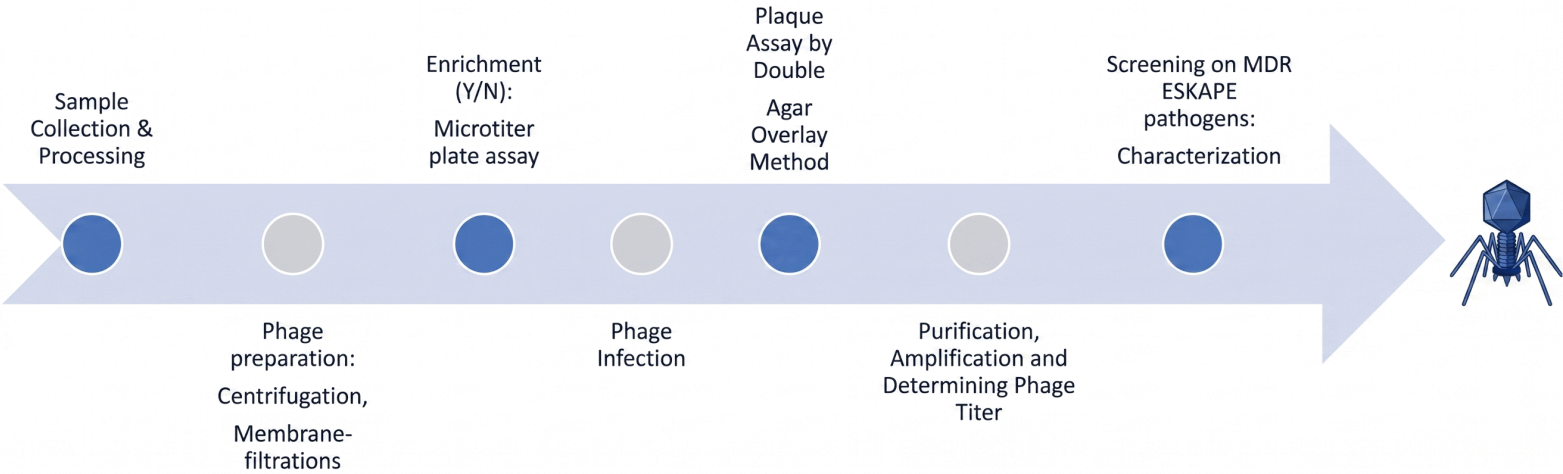
A flow chart, summarizing step-wise isolation of bacteriophages.

Bacteriophages (phages for short) targeting multidrug-resistant (MDR) bacterial isolates will be isolated from sources such as soil, sewage, decomposing organic material, and residual microbial culture supernatants. A flow chart, summarizing step-wise isolation of bacteriophages is given in Fig 2. For isolation, plaque purification, and amplification of phages, the Double Agar Overlay Method would be adopted with minor variations [8,9]. Each sample will be mixed thoroughly with Phage Buffer (100 mM Tris-HCl, pH 7.5; 100 mM MgSO₄; 1 mM CaCl₂; 68 mM NaCl) and allowed to settle. The liquid phase will be carefully transferred to a fresh collection tube, then centrifuged at 10,000 rpm for 10 min. The supernatant will be syringe-filtered through a 0.45 µm membrane filter. This filtrate is a phage lysate that will be used for phage isolation.

#### Phage enrichment.

To enrich phages against target bacteria, the bacterial culture (OD₆₀₀ = 0.4–0.6) and nutrient media (Luria Broth) will be added to the phage lysate and incubated at 37 °C with gentle shaking. Overnight incubation will be followed by centrifugation at 10,000 rpm for 10 minutes. The supernatant obtained will be then used for phage isolation, using the Double Agar Overlay Method [10]. Briefly, for phage infection of target bacteria, a mixture of 100 µL of mid-log bacterial culture (OD₆₀₀ = 0.4–0.6) and 100 µL of phage lysate will be incubated at room temperature for 30–45 min and then overlaid with 4.5 mL of molten soft agar (containing 1 mM CaCl₂) on LB agar plates. Plates will be incubated at 37 °C for 16-24 h to allow plaque formation. Individual plaques will be picked using sterile tips and purified through at least three successive rounds of single-plaque isolation. Samples can also be directly screened for phage isolation without the enrichment step, although fewer phages might be obtained.

#### Phage amplification.

Phage amplification will be carried out by plating purified phages at dilutions that produce web-like plaque patterns with high plaque density. Multiple such plates will be flooded with 4–5 mL phage buffer and incubated at 4 °C for 4 h (or overnight) to allow phage diffusion into the buffer. The resulting high-titre lysates will be collected, centrifuged at 8,000 rpm for 10 min to remove bacterial debris, and the supernatant will be syringe filter-sterilized (0.22 µm).

#### Phage purification by PEG precipitation and titre determination.

For further purification and high-titre (~10⁹-10¹² PFU/mL) phage preparation, filtered lysates will be treated with PEG 8000 (20%, NaCl 15%), mixed thoroughly, and incubated at 4 °C [11]. To collect the precipitate, samples will be centrifuged at 13,000 × g for 20 min at 4 °C, and the resulting pellet will be washed once with Phage Buffer to remove residual PEG and resuspended in 3–4 mL of SM Buffer (5.8 g NaCl, 2 g MgSO₄·7H₂O, 50 mL 1 M Tris-HCl pH 7.5, per Litre) (Bonilla et al., 2016). To allow complete dissolution, the suspension will be incubated at room temperature for 30 min, then gently pipetted. After centrifugation (13,000 × g, 10 min), the clarified supernatant will be stored at 4 °C, and its titer (PFU/mL) will be determined.

#### Phage screening using Reasazurin Assay.

For medium throughput screening, phage lysates can also be screened using a resazurin-based colourimetric assay [12]. To perform this assay, bacterial cultures and phage lysate (enriched or unenriched) will be added to 96-well microtiter plates and incubated at 37 °C. After 24 h, the reagent will be added to a final concentration of 0.1% (v/v), mixed gently, and incubated in the dark for 1 h. A pink colour indicates the presence of viable/metabolically active cells (no phage activity), whereas a blue colour indicates phage-induced bacterial lysis. The controls included in the assay will be:

- Positive Control: Bacterial cells mixed with a known lytic phage
- Negative Control: Bacterial cells without phage lysate
- Blank: Only culture medium

All treatments will be performed in triplicate. Phages will be isolated from the wells showing lysis by plaque assay and purified by PEG precipitation as described above. In addition, endotoxin levels in purified phage preparations will be quantified using the Limulus Amebocyte Lysate (LAL) assay, and sterility testing will be performed to ensure the absence of viable bacterial contamination, thereby confirming the safety and purity of the preparations before downstream applications such as therapeutic evaluation [13].

### Phage characterization

#### Morphology and stability testing.

To determine phage morphology, Transmission Electron Microscopy (TEM) will be performed**.** Purified phage suspensions (~10⁹-10¹⁰ PFU/mL) will be adsorbed onto carbon-coated copper grids, negatively stained with 2% (w/v) uranyl acetate (pH 7.0), and visualised using a JEOL TEM 1400 operated at 120 kV, with images captured at 30,000 × magnification [14]

#### Temperature and pH stability.

Phage stability will be assessed under varying pH, temperature, and UV exposure conditions. Aliquots of purified phage preparation (100 µl; 10^9^/10^10^ PFU/ml) will be incubated at a range of temperatures and pH for 1 h. For pH stability, phage preparations (100 µl; 10^9^/10^10^ PFU/ml) will be diluted in suitable buffers, adjusted to pH values ranging from 2 to 8. Post-incubation, phage titres will be determined to assess viability across the tested conditions [15].

#### Storage.

Long-term storage will be carried out either at 4 °C in SM buffer supplemented with chloroform or at –80 °C in glycerol stocks, while lyophilization may also be employed for extended preservation [16]. Before conducting downstream assays, phage viability will be determined by periodically titrating stocks by plaque assay.

### DNA extraction

The Quick-DNA™ Fecal/Soil Microbe Midiprep Kit (Zymo Research, USA; Catalog No. D6110) will be used to extract high-quality, PCR-ready DNA from culture as well as human, livestock and environmental samples. Samples will be lysed in ZR BashingBead™ Lysis/Filtration Tubes using bead beating, followed by purification with Zymo-Spin™ Technology and filtration to remove inhibitors. DNA will be eluted and further filtered to ensure purity for downstream applications.

Nucleic acid quality will be assessed prior to library preparation. DNA concentration will be measured using Qubit HS assays (Thermo Fisher Scientific, #Q32854) and integrity will be checked via E-Gel™ Power Snap Electrophoresis System (Invitrogen Catalog No G8300).

For Phage Genomic DNA isolation, Phenol-Chloroform-Isoamyl alcohol (PCI) DNA extraction protocol will be carried out to isolate phage genomic DNA. To remove contaminating bacterial nucleic acids from the phage preparation [17], 12.5 mM MgCl₂, 0.8 mU/mL DNase I, and 0.01 μg/μL RNase will be added to the phage lysate (10⁹ pfu/mL), followed by the addition of 20 mM EDTA, 50 μg/μL Proteinase K, and 0.5% SDS. The mixture will be incubated at 55 °C for one hour with vortexing every 20 minutes. After incubation, the lysate will be mixed with an equal volume of PCI (25:24:1) and centrifuged at 13,000 rpm for 15 minutes to extract the aqueous layer. This extraction will be repeated three times, with the final step performed using chloroform.

The DNA will then be precipitated by adding 0.15 M sodium acetate and chilled 95% ethanol and stored at –20 °C. Precipitated DNA will be collected by centrifugation under the same conditions, washed with 70% ethanol, dried at room temperature, and resuspended in nuclease-free water. The quality and purity of DNA will be assessed by agarose gel electrophoresis and visualized under UV light after ethidium bromide staining. The A260:A280 and A260:A230 ratios will be determined using a Nanodrop or Qubit fluorometer.

### Whole-genome sequencing, metagenomic sequencing, and bioinformatics analysis

Whole-genome sequencing (WGS) will be performed at a certified high-throughput sequencing facility MedGenome Labs Pvt. Ltd., Bengaluru, India, using the Illumina NovaSeq 6000 platform. Sequencing will be carried out using an S4 flow cell, generating paired-end reads (2 × 150 bp). A minimum sequencing depth of ≥30 × will be targeted for bacterial genomes, while higher coverage (≥100×) will be used for bacteriophage genomes to ensure accurate assembly. Library preparation and sequencing will be carried out according to standard Illumina protocols as implemented at the sequencing facility.

Raw sequencing reads will undergo quality assessment using FastQC (v0.11.9) [18] and trimming using fastp (v0.23.2) [19]. De novo genome assembly for bacterial isolates will be performed using SPAdes (v3.15.5) [20], and assembly quality will be evaluated using QUAST (v5.2.0) [21]. Genome annotation will be conducted using Prokka (v1.14.6) [22] and RASTtk [23], with additional functional annotation using BLAST+ (v2.13.0) [24] and HMMER (v3.3.2) [25]. AMR genes will be identified using ResFinder (v4.1) [26]. All tools will be used with default parameters unless otherwise specified.

To investigate genomic relatedness and transmission dynamics, core genome single nucleotide polymorphisms (SNPs) will be identified using Snippy (v4.6.0) [27], recombination regions will be removed using Gubbins (v3.2.1) [28], and phylogenetic trees will be constructed using IQ-TREE (v2.2.0) [29]. Plasmid and mobile genetic element analysis will be performed using PlasmidFinder (v2.1) [30] and MOB-suite (v3.1.0) [31].

### Metagenomic sequencing (mNGS) and functional profiling

In addition to isolate-based WGS, metagenomic next-generation sequencing (mNGS) will be performed on selected human, livestock, and environmental samples to characterize the broader microbial community and resistome. DNA extracted directly from samples will be sequenced on the Illumina NovaSeq 6000 platform, generating paired-end reads (2 × 150 bp).

Raw metagenomic reads will be quality-filtered using FastQC [18] and fastp [19]. Host-derived reads will be removed using Bowtie2 (v2.5.1) [32] by mapping against appropriate reference genomes. Cleaned reads will be assembled using metaSPAdes (v3.15.5) [33]. Taxonomic profiling will be performed using Kraken2 (v2.1.2) [34] and Bracken [35], and microbial diversity will be assessed using standard ecological indices.

Functional resistome profiling will be conducted using the nf-core/funcscan pipeline (v3.0.0) [36], a reproducible Nextflow-based workflow that integrates tools such as DeepARG [37] for AMR gene detection and generates standardized outputs using MultiQC [38].

### Bacteriophage genome sequencing and assembly

Whole Genome Sequencing will be performed on the Illumina NovaSeq 6000 platform to generate paired-end reads (2 × 150 bp), targeting a sequencing depth of ≥100 × . Raw reads will be quality-checked using FastQC [18] and trimmed using fastp [19].

De novo assembly of phage genomes will be performed using SPAdes (v3.15.5) [20] in --careful mode, with multiple k-mer sizes (e.g., 21, 33, 55, 77). For complex datasets, metaSPAdes [33] may be used. Assembly graphs will be visualized using Bandage (v0.8.1) [39] to confirm genome completeness and circularization where applicable.

Phage identification and completeness assessment will be performed using VirSorter2 [40] and PHASTER [41]. Genome annotation will be conducted using Prokka [22] and cross-validated using RASTtk [23], with further functional annotation using BLAST+ [24] and HMMER [25].

To ensure suitability for therapeutic applications, phage genomes will be systematically screened for the absence of lysogeny-associated genes, virulence factors, and antimicrobial resistance determinants. Antimicrobial resistance genes will be identified using ResFinder [26] and the Comprehensive Antibiotic Resistance Database (CARD, v3.2.6) [42] using default parameters, supplemented by manual curation. Virulence-associated genes will be identified using the Virulence Factor Database (VFDB) [43] and screened using ABRicate (v1.0.1) [44]. Only strictly lytic phages devoid of undesirable genetic elements will be selected for downstream applications against antimicrobial-resistant bacterial pathogens. The assembled sequencing data of phages in FASTA format, suggestive of only strictly lytic phages that lack these undesirable elements will be advanced for consideration as therapeutic candidates [45]. Final selection of therapeutic phages will be based on multiple criteria: absence of lysogeny, virulence, or AMR-associated genes; high efficiency of plating (EOP ≥ 0.01) on target MDR isolates; stability during storage and relevant physiological conditions; and host range type.

### Data analysis

Descriptive statistics will be used to summarize AMR prevalence across human, animal, and environmental samples. Bioinformatics pipelines will be applied to metagenomic datasets to profile resistome and phage diversity, with diversity indices (e.g., Shannon, Simpson) used to quantify gene richness and evenness. Statistical analyses will be performed using MedCalc or R, with a p-value of <0.05 considered statistically significant.

To investigate potential animal-to-human and inter-reservoir transmission, WGS data from bacterial isolates will be analyzed using a standardized SNP-based phylogenetic pipeline. Core genome SNPs will be identified using Snippy, with recombination regions excluded using Gubbins, and phylogenetic trees constructed with IQ-TREE. Genomic relatedness thresholds (e.g., ≤ 10 SNPs difference) will be used to infer putative transmission events. In addition, plasmid and mobile genetic element (MGE) tracking will be conducted using tools such as PlasmidFinder and MOB-suite to assess horizontal gene transfer of resistance determinants across species and reservoirs. Comparisons will be made between isolated genomes of humans, livestock, and environmental samples to identify shared clones or plasmids. Metagenomic resistome data will be integrated with isolate-based WGS results to provide complementary evidence of AMR gene flow within and between reservoirs.

### Ethics and dissemination

Ethical approval for collection of human, livestock, and environmental samples will be obtained from the Institutional Ethics Committee (IEC) of CIIMS, the Institutional Biosafety Committee (IBSC) of CSIR-NEERI, and the Institutional Animal Ethics Committee (IAEC) of NVC, MAFSU. In addition, necessary permissions will be secured from local veterinary authorities for animal sampling. Written informed consent will be obtained from all human participants, with study objectives, procedures, risks, and benefits explained in the local language. Participant confidentiality will be safeguarded through anonymized datasets. All laboratory procedures will comply with biosafety guidelines, with MDR bacterial isolates handled in BSL-2 facilities as appropriate, and phage isolation work conducted under IBSC oversight. Study findings will be disseminated through peer-reviewed publications, international scientific conferences, and deposition of genomic and metagenomic datasets in public repositories such as National Center for Biotechnology Information (NCBI) and European Nucleotide Archive (ENA).

## Discussion

This study represents a comprehensive One Health investigation that integrates human, animal, and environmental health to better understand the prevalence, dissemination, and control of AMR farm-exposed populations in Nagpur, India. By combining longitudinal sampling with high-throughput sequencing and bacteriophage characterization, the study will generate novel datasets and identify scalable phage therapy candidates, providing actionable insights to inform national AMR control policies.

AMR remains a significant public health challenge in India, contributing to high morbidity and mortality rates, particularly among children [46]. The rapid emergence and dissemination of resistant bacteria, including resistance to last-resort antibiotics such as carbapenems and colistin, has intensified the public health concern [46,47]. Animal farms, where humans, livestock, and environmental reservoirs closely interact, constitute critical yet understudied ecosystems for the circulation of AMR bacteria and ARGs [48,49]. Despite their importance, systematic investigations into AMR prevalence in livestock and associated human populations in India are limited, with most evidence derived from small, localized studies [48]. The unregulated use of antibiotics in livestock, combined with the absence of comprehensive surveillance systems, further exacerbates the risk and highlights the need for integrated monitoring [49]. Mastitis in dairy cattle and buffaloes, a common clinical problem, has been shown to harbor diverse bacterial populations, including multidrug-resistant strains, underscoring livestock as an important reservoir of AMR [50].

Bacteriophages offer a promising alternative or complementary approach to conventional antibiotics, particularly against multidrug-resistant pathogens [47,51]. Phages are host-specific, self-replicating, environmentally sustainable, and generally safe for humans and animals [47]. Advances in next-generation sequencing and genomic annotation allow systematic identification and characterization of phages, facilitating their potential therapeutic use [47,51]. Previous studies have demonstrated that phage therapy can effectively target drug-resistant bacteria, disrupt biofilms, and even interfere with the transfer of AMR plasmids [51,52]. The longitudinal design of this study, including sampling across multiple seasons, will capture temporal fluctuations in AMR prevalence and ARG dissemination, offering insights into the influence of environmental and climatic factors on microbial ecology [46,49]. Detailed metadata collection—including human demographics, comorbidities, antibiotic exposure, and livestock management practices—will support robust analyses of risk factors for interspecies AMR transmission [49]. Environmental physicochemical parameters, such as pH, dissolved oxygen, and heavy metal concentrations, will be assessed to understand their impact on microbial communities and the persistence of resistance genes [27].

The anticipated outputs of this study include a comprehensive dataset on longitudinal AMR prevalence and ARG distribution across human, livestock, and environmental reservoirs, a biobank of AMR bacterial strains and therapeutic bacteriophages, high-quality phage genome sequences annotated for therapeutic suitability, evidence to guide AMR stewardship programs and policy interventions, and a foundation for a multicentric national AMR surveillance and phage therapy program. The strengths of this study include its comprehensive and multidisciplinary design, integration of human, animal, and environmental data, use of next-generation sequencing technologies, and collaborative expertise. Limitations include the absence of pre-existing baseline prevalence data, the lack of hypothesis-driven statistical modelling, and potential challenges in participant retention over the longitudinal study period. Overall, this study will generate critical insights into the dynamics of AMR, inform One Health strategies, and support the development of sustainable interventions, including phage-based therapeutics, to protect human, animal, and environmental health.

## Conclusion

This study protocol outlines a comprehensive, longitudinal investigation into the prevalence, dissemination, and control of AMR bacteria and ARGs across livestock, humans, and environmental reservoirs in dairy farms in Nagpur, India. By integrating microbial isolation, antimicrobial susceptibility testing, bacteriophage isolation and characterization, and metagenomic profiling under a One Health framework, the study aims to generate critical insights into the ecological and therapeutic aspects of AMR. The results are expected to identify potential bacteriophage candidates for future therapeutic applications, reveal seasonal and geographic patterns in AMR dissemination, and provide evidence-based guidance for interventions to mitigate AMR. Ultimately, this work will contribute to a better understanding of AMR dynamics and the development of sustainable strategies to protect human, animal, and environmental health.

Building on these findings, future research will focus on expanding surveillance networks across varied geographic regions and incorporating socio-economic factors influencing AMR spread. Furthermore, the data generated will inform national and regional policy frameworks geared toward antimicrobial stewardship, regulation of antibiotic usage in agriculture, and promotion of phage therapy as a complementary approach to combat multidrug-resistant infections. This integrated knowledge will be pivotal in shaping One Health policies and strategic interventions aimed at curbing the AMR crisis in India and similar settings globally.

## Abbreviations

AMR: Antimicrobial Resistance
ARG: Antimicrobial Resistance Gene
AST: Antimicrobial Susceptibility Testing
BSL: Biosafety Level
CARD: Comprehensive Antibiotic Resistance Database
CFU: Colony Forming Units
CT-SMAC: Cefixime-Tellurite Sorbitol MacConkey Agar
DNA: Deoxyribonucleic Acid
EDTA: Ethylenediaminetetraacetic Acid
EMB: Eosin Methylene Blue Agar
EOP: Efficiency of Plating
Gubbins: Genealogies Unbiased By recombination’s In Nucleotide Sequences
HiCrome: A Chromogenic Culture Medium Brand
IBSC: Institutional Biosafety Committee
IEC: Institutional Ethics Committee
IAEC: Institutional Animal Ethics Committee
LAL: Limulus Amebocyte Lysate (assay)
MIC: Minimum Inhibitory Concentration
MDR: Multidrug Resistant
MGE: Mobile Genetic Element
mNGS: Metagenomic Next-Generation Sequencing
NCBI: National Center for Biotechnology Information
PCR: Polymerase Chain Reaction
PFU: Plaque Forming Units
PCI: Phenol-Chloroform-Isoamyl Alcohol
PPE: Personal Protective Equipment
qPCR: Quantitative Polymerase Chain Reaction
RAST: Rapid Annotation using Subsystem Technology
RNA: Ribonucleic Acid
RGI: Resistance Gene Identifier
SNP: Single Nucleotide Polymorphism
SPAdes: St. Petersburg genome assembler
TEM: Transmission Electron Microscopy
WGS: Whole Genome Sequencing
