# Supplementary material for: Study protocol on antimicrobial resistance burden, transmission dynamics, and therapeutic bacteriophages in livestock and exposed farming populations in Nagpur, India: An integrated One Health approach
Source: PLoS One. 2026 Jun 16;21(6):e0350919. doi: 10.1371/journal.pone.0350919 (PMC13271485; doi:10.1371/journal.pone.0350919)
Supplement: Attachment — Submitted filename: Response to Reviewers.pdf [file pone.0350919.s002.pdf]

<u>Comment 1</u>: The facility that will be performing the whole genome sequencing, the type of flow cell, and the type of read pairs

Response: We thank the reviewer for highlighting this important point**.** Whole-genome sequencing (WGS) will be performed at MedGenome Labs Pvt. Ltd., Bengaluru, India a certified high-throughput sequencing facility using the **Illumina NovaSeq 6000 platform**. Sequencing will be carried out using an **S4 flow cell**, generating **paired-end reads of 150–151 bp (2 × 150/151 bp)**. A minimum sequencing depth of ≥30× will be targeted for bacterial genomes, while higher coverage (≥100×) will be used for bacteriophage genomes to ensure accurate assembly.

<u>Comment 2</u>: The version of the tools that will be used and appropriate citations for those tools

Response: We thank the reviewer for this valuable suggestion. Bioinformatics analyses will be performed using well-established and version-controlled tools. Raw sequencing reads will be quality assessed using **FastQC (v0.11.9)** and trimmed using **fastp (v0.23.2)**. De novo genome assembly will be conducted using **SPAdes (v3.15.5)**, and assembly quality will be evaluated using **QUAST (v5.2.0)**. Genome annotation will be performed using **Prokka (v1.14.6)** and **RASTtk**. Antimicrobial resistance genes will be identified using **ResFinder (v4.1)**, and sequence similarity searches will be conducted using **BLAST+ (v2.13.0)**. Protein domain analysis will be performed using **HMMER (v3.3.2)**. Phylogenetic analyses will be carried out using **Snippy (v4.6.0)**, **Gubbins (v3.2.1)**, and **IQ-TREE (v2.2.0)**. Functional resistome profiling will be performed using the **nf-core/funcscan pipeline (v3.0.0)**. All tools will be used with default parameters unless otherwise specified.

Appropriate citations for all tools have been included in the revised manuscript.

<u>Comment 3</u>: Details regarding phage genome assembly including the tools that will be used, version, flags

Response: We thank the reviewer for this insightful comment. Bacteriophage genome assembly will be performed using **SPAdes (v3.15.5)** in **--careful mode** to minimize assembly errors, with multiple k-mer sizes (e.g., 21, 33, 55, 77). For complex datasets, **metaSPAdes** may be used. Raw sequencing reads will be quality-checked using **FastQC (v0.11.9)** and trimmed using **fastp (v0.23.2)** prior to assembly. Assembly graphs will be visualized using **Bandage (v0.8.1)** to confirm genome completeness and potential circularization.

Phage identification and completeness assessment will be conducted using **VirSorter2** and **PHASTER**. Genome annotation will be performed using **Prokka (v1.14.6)** and cross-validated using **RASTtk**, with further functional annotation using **BLAST+ (v2.13.0)** and **HMMER (v3.3.2)**. To ensure suitability for therapeutic applications, phage genomes will be screened for the absence of lysogeny-associated genes, virulence factors, and antimicrobial resistance genes using **ResFinder**, **CARD**, and manual curation.
