# Supplementary material for: Study protocol on antimicrobial resistance burden, transmission dynamics, and therapeutic bacteriophages in livestock and exposed farming populations in Nagpur, India: An integrated One Health approach
Source: PLoS One. 2026 Jun 16;21(6):e0350919. doi: 10.1371/journal.pone.0350919 (PMC13271485; doi:10.1371/journal.pone.0350919)
Supplement: Peer review history

# Editor report: Decision Letter 0

**DOI:** 10.1371/journal.pone.0350919.r001

**Authors:** Katherine Kokkinias

8 Apr 2026

PONE-D-26-06953

Study Protocol on Antimicrobial Resistance Burden, Transmission Dynamics, and Therapeutic Bacteriophages in Livestock and Exposed Farming Populations in Nagpur, India: An Integrated One Health Approach.

PLOS One

Dear Dr. Kashyap,

Thank you for submitting your manuscript to PLOS ONE. After careful consideration, we feel that it has merit but does not fully meet PLOS ONE’s publication criteria as it currently stands. Therefore, we invite you to submit a revised version of the manuscript that addresses the points raised during the review process.

==============================

Upon reviewing your manuscript, we have a few additional requests regarding the reporting for whole genome sequencing. Please include the following information:

1. The facility that will be performing the whole genome sequencing, the type of flow cell, and the type of read pairs (i.e. 151-bp paired-end reads)
2. The version of the tools that will be used and appropriate citations for those tools
3. Details regarding phage genome assembly including the tools that will be used, version, flags

==============================

Please submit your revised manuscript by May 22 2026 11:59PM. If you will need more time than this to complete your revisions, please reply to this message or contact the journal office at plosone@plos.org. When you're ready to submit your revision, log on to https://www.editorialmanager.com/pone/ and select the 'Submissions Needing Revision' folder to locate your manuscript file.

Please include the following items when submitting your revised manuscript:

- A letter that responds to each point raised by the academic editor and reviewer(s). You should upload this letter as a separate file labeled 'Response to Reviewers'.
- A marked-up copy of your manuscript that highlights changes made to the original version. You should upload this as a separate file labeled 'Revised Manuscript with Track Changes'.
- An unmarked version of your revised paper without tracked changes. You should upload this as a separate file labeled 'Manuscript'.

If you would like to make changes to your financial disclosure, please include your updated statement in your cover letter. Guidelines for resubmitting your figure files are available below the reviewer comments at the end of this letter.

If applicable, we recommend that you deposit your laboratory protocols in protocols.io to enhance the reproducibility of your results. Protocols.io assigns your protocol its own identifier (DOI) so that it can be cited independently in the future. For instructions see: https://journals.plos.org/plosone/s/submission-guidelines#loc-laboratory-protocols. Additionally, PLOS ONE offers an option for publishing peer-reviewed Lab Protocol articles, which describe protocols hosted on protocols.io. Read more information on sharing protocols at https://plos.org/protocols?utm_medium=editorial-email&utm_source=authorletters&utm_campaign=protocols.

As the corresponding author, your ORCID iD is verified in the submission system and will appear in the published article. PLOS supports the use of ORCID, and we encourage all coauthors to register for an ORCID iD and use it as well. Please encourage your coauthors to verify their ORCID iD within the submission system before final acceptance, as unverified ORCID iDs will not appear in the published article. *Only*  the individual author can complete the verification step; PLOS staff *cannot*  verify ORCID iDs on behalf of authors.

We look forward to receiving your revised manuscript.

Kind regards,

Katherine Demi Kokkinias, Ph.D.

Staff Editor

PLOS One

Journal requirements:

When submitting your revision, we need you to address these additional requirements.

1. Please ensure that your manuscript meets PLOS ONE's style requirements, including those for file naming. The PLOS ONE style templates can be found at

https://journals.plos.org/plosone/s/file?id=wjVg/PLOSOne_formatting_sample_main_body.pdf and

https://journals.plos.org/plosone/s/file?id=ba62/PLOSOne_formatting_sample_title_authors_affiliations.pdf

If the reviewer comments include a recommendation to cite specific previously published works, please review and evaluate these publications to determine whether they are relevant and should be cited. There is no requirement to cite these works unless the editor has indicated otherwise.

Please review your reference list to ensure that it is complete and correct. If you have cited papers that have been retracted, please include the rationale for doing so in the manuscript text, or remove these references and replace them with relevant current references. Any changes to the reference list should be mentioned in the rebuttal letter that accompanies your revised manuscript. If you need to cite a retracted article, indicate the article’s retracted status in the References list and also include a citation and full reference for the retraction notice.

[Note: HTML markup is below. Please do not edit.]

To ensure your figures meet our technical requirements, please review our figure guidelines: https://journals.plos.org/plosone/s/figures

You may also use PLOS’s free figure tool, NAAS, to help you prepare publication quality figures: https://journals.plos.org/plosone/s/figures#loc-tools-for-figure-preparation.

NAAS will assess whether your figures meet our technical requirements by comparing each figure against our figure specifications.

# Author comment: Author response to Decision Letter 1

**DOI:** 10.1371/journal.pone.0350919.r002

19 May 2026

Comment 1: The facility that will be performing the whole genome sequencing, the type of flow cell, and the type of read pairs

Response: We thank the reviewer for highlighting this important point. Whole-genome sequencing (WGS) will be performed at MedGenome Labs Pvt. Ltd., Bengaluru, India a certified high-throughput sequencing facility using the Illumina NovaSeq 6000 platform. Sequencing will be carried out using an S4 flow cell, generating paired-end reads of 150–151 bp (2 × 150/151 bp). A minimum sequencing depth of ≥30× will be targeted for bacterial genomes, while higher coverage (≥100×) will be used for bacteriophage genomes to ensure accurate assembly.

Comment 2: The version of the tools that will be used and appropriate citations for those tools

Response: We thank the reviewer for this valuable suggestion. Bioinformatics analyses will be performed using well-established and version-controlled tools. Raw sequencing reads will be quality assessed using FastQC (v0.11.9) and trimmed using fastp (v0.23.2). De novo genome assembly will be conducted using SPAdes (v3.15.5), and assembly quality will be evaluated using QUAST (v5.2.0). Genome annotation will be performed using Prokka (v1.14.6) and RASTtk. Antimicrobial resistance genes will be identified using ResFinder (v4.1), and sequence similarity searches will be conducted using BLAST+ (v2.13.0). Protein domain analysis will be performed using HMMER (v3.3.2). Phylogenetic analyses will be carried out using Snippy (v4.6.0), Gubbins (v3.2.1), and IQ-TREE (v2.2.0). Functional resistome profiling will be performed using the nf-core/funcscan pipeline (v3.0.0). All tools will be used with default parameters unless otherwise specified.

Appropriate citations for all tools have been included in the revised manuscript.

Comment 3: Details regarding phage genome assembly including the tools that will be used, version, flags

Response: We thank the reviewer for this insightful comment. Bacteriophage genome assembly will be performed using SPAdes (v3.15.5) in --careful mode to minimize assembly errors, with multiple k-mer sizes (e.g., 21, 33, 55, 77). For complex datasets, metaSPAdes may be used. Raw sequencing reads will be quality-checked using FastQC (v0.11.9) and trimmed using fastp (v0.23.2) prior to assembly. Assembly graphs will be visualized using Bandage (v0.8.1) to confirm genome completeness and potential circularization.

Phage identification and completeness assessment will be conducted using VirSorter2 and PHASTER. Genome annotation will be performed using Prokka (v1.14.6) and cross-validated using RASTtk, with further functional annotation using BLAST+ (v2.13.0) and HMMER (v3.3.2). To ensure suitability for therapeutic applications, phage genomes will be screened for the absence of lysogeny-associated genes, virulence factors, and antimicrobial resistance genes using ResFinder, CARD, and manual curation.

Attachment Submitted filename: Response to Reviewers.pdf

# Editor report: Decision Letter 1

**DOI:** 10.1371/journal.pone.0350919.r003

**Authors:** Katherine Kokkinias

21 May 2026

Study Protocol on Antimicrobial Resistance Burden, Transmission Dynamics, and Therapeutic Bacteriophages in Livestock and Exposed Farming Populations in Nagpur, India: An Integrated One Health Approach.

PONE-D-26-06953R1

Dear Dr. Kashyap,

As a Peer-Reviewed Funded Protocol, your submission is eligible for expedited review by in-house editors. Based on our evaluation, we are satisfied that your manuscript meets our publication criteria for Study Protocols, and is therefore considered to be suitable for publication subject to final journal requirements

Within one week, you’ll receive an e-mail detailing the required amendments. When these have been addressed, you’ll receive a formal acceptance letter and your manuscript will be scheduled for publication.

An invoice will be generated when your article is formally accepted. Please note, if your institution has a publishing partnership with PLOS and your article meets the relevant criteria, all or part of your publication costs will be covered. Please make sure your user information is up-to-date by logging into Editorial Manager at Editorial Manager® and clicking the ‘Update My Information' link at the top of the page. For questions related to billing, please contact billing support.

If your institution or institutions have a press office, please notify them about your upcoming paper to help maximize its impact. If they’ll be preparing press materials, please inform our press team as soon as possible -- no later than 48 hours after receiving the formal acceptance. Your manuscript will remain under strict press embargo until 2 pm Eastern Time on the date of publication. For more information, please contact onepress@plos.org.

Kind regards,

Katherine Demi Kokkinias, Ph.D.

Staff Editor

PLOS One

Additional Editor Comments (optional):

Reviewers' comments:

# Editor report: Acceptance letter

**DOI:** 10.1371/journal.pone.0350919.r004

**Authors:** Katherine Kokkinias

PONE-D-26-06953R1

PLOS One

Dear Dr. Kashyap,

I'm pleased to inform you that your manuscript has been deemed suitable for publication in PLOS One. Congratulations! Your manuscript is now being handed over to our production team.

At this stage, our production department will prepare your paper for publication. This includes ensuring the following:

* All references, tables, and figures are properly cited

* All relevant supporting information is included in the manuscript submission,

* There are no issues that prevent the paper from being properly typeset

You will receive further instructions from the production team, including instructions on how to review your proof when it is ready. Please keep in mind that we are working through a large volume of accepted articles, so please give us a few days to review your paper and let you know the next and final steps.

Lastly, if your institution or institutions have a press office, please let them know about your upcoming paper now to help maximize its impact. If they'll be preparing press materials, please inform our press team within the next 48 hours. Your manuscript will remain under strict press embargo until 2 pm Eastern Time on the date of publication. For more information, please contact onepress@plos.org.

You will receive an invoice from PLOS for your publication fee after your manuscript has reached the completed accept phase. If you receive an email requesting payment before acceptance or for any other service, this may be a phishing scheme. Learn how to identify phishing emails and protect your accounts at https://explore.plos.org/phishing.

If we can help with anything else, please email us at customercare@plos.org.

Thank you for submitting your work to PLOS ONE and supporting open access.

Kind regards,

PLOS ONE Editorial Office Staff

on behalf of

Dr. Katherine Demi Kokkinias

Staff Editor

PLOS One
